# Determination of Crucial Immunogenic Epitopes in Major Peanut Allergy Protein, Ara h2, via Novel Nanoallergen Platform

**DOI:** 10.1038/s41598-017-04268-6

**Published:** 2017-06-21

**Authors:** Peter E. Deak, Maura R. Vrabel, Tanyel Kiziltepe, Basar Bilgicer

**Affiliations:** 1 Department of Chemical and Biomolecular Engineering, University of Notre Dame, Notre Dame, IN 46556 USA; 2 Advanced Diagnostics and Therapeutics, University of Notre Dame, Notre Dame, IN 46556 USA; 3 Department of Chemistry and Biochemistry, University of Notre Dame, Notre Dame, IN 46556 USA

## Abstract

Current methods for detection and diagnosis of allergies do not provide epitope specific immunogenic information and hence lack critical information that could aid in the prediction of clinical responses. To address this issue, we developed a nanoparticle based platform, called nanoallergens that enable multivalent display of potential allergy epitopes for determining the immunogenicity of each IgE binding epitope. By synthesizing nanoallergens that present various epitopes from the major peanut allergen, Ara h2, we directly determined the immunogenicity of each epitope, alone and in combination with other epitopes, using patient sera. This information provided insights on which epitopes are most critical for physiological responses to Ara h2 and revealed the importance of both high and low affinity epitopes for allergic responses. We anticipate the nanoallergen platform to be used to provide information regarding allergic reactions and therefore potentially aid in more accurate diagnosis and design of personalized treatment options.

## Introduction

Food allergies are a type I hypersensitivity (allergy) to proteins within certain foods that can result in symptoms ranging from harmless skin irritation to a life-threatening anaphylactic reaction. Type I hypersensitivity is caused when an allergen binds multivalently to allergen specific IgEs bound to Fc epsilon receptors (FcεRIs) present on the surface of mast cells or basophils, causing receptor clustering and culminating in degranulation. While only 3% percent of adults suffer from food allergies, 8% percent of children under four years of age have a food allergy^1, 2^. Consequently, food allergies are a rapidly growing problem in developed nations, resulting in a dire need for more accurate and information-rich diagnostic testing and therapeutic options^3, 4^. Effective allergy tests should provide a measure of how strongly an allergen stimulates a cellular immune response (immunogenicity) and how the intensity of this cellular response correlates to physiological symptoms. Allergy tests can be divided into two broad categories: (1) clinical allergy testing such as the skin prick test (SPT) which uses allergens to stimulate a controlled response on a patient and (2) *in vitro* assays that evaluate the level of allergen or epitope specific IgE (sIgE) antibodies in patients’ sera. The SPT test provides qualitative measurement for the intensity of the local degranulation, but has poor specificity when predicting the severity of physiological responses to allergen and cannot specifically test individual allergen epitopes. Recently, microarrays have been used to identify food allergen sIgEs in clinical samples not only to a specific allergen protein, but also to the allergen epitopes by generating linear peptides as epitope mimetics^5–9^. This information, while useful in evaluating the binding of major IgE epitopes, does not present any information regarding degranulation responses or how important these epitopes are for overall physiological reactions to allergens. This is because binding affinity can only provide a relative measure of immunogenicity and does not directly correlate, given the complexities of multivalent binding^10^. Immunogenicity also depends upon multivalent interactions between multiple IgEs and epitopes as well as intracellular regulatory responses and underestimates the contributions of moderate to low affinity epitopes^11, 12^. Given these limitations, novel methods that can directly predict the clinical and physiological severity of an allergic reactionwould greatly improve both the current understanding of allergen immunogenicity.

In order to address the weakness of current allergy testing methods, this manuscript describes a novel nanoparticle platform, called nanoallergens, that can individually determine the immunogenicity of each IgE binding epitope of an allergen^13^. Liposomal nanoparticles have been used for many decades as drug delivery vehicles and for active targeting of diseased cells^14, 15^. In our recent studies, we have developed a novel method for precise loading and efficient display of peptides on a liposomal surface. This method, combined with the nanoallergen platform enables a reliable means to incorporate one or more allergen epitopes on the same nanoparticle surface with a high degree of control over particle size and epitope stoichiometry^16–18^. As a result, nanoallergens can efficiently display potential IgE binding epitopes in a multivalent fashion and provide a novel and practical experimental model system to dissect epitope immunogenicity. In the past, liposomes have been employed as carriers for allergen proteins, either to aid sensitization or to prevent allergic reactions from pre-sensitized mice^19, 20^. Nevertheless, liposomes have never been used to present individual epitopes from allergen proteins to trigger allergic reactions. In this manuscript, we introduce nanoallergens as a versatile platform to directly determine individual epitope immunogenicity for a particular patient by systematically evaluating the epitopes of the major peanut allergen, Ara h2, using clinical samples. This epitope specific information would provide a clearer picture of allergen potency, allowing more precise clinical diagnoses and potentially invaluable information about which epitopes future therapeutics should target.

## Results

### Design, Synthesis and Characterization of Nanoallergens

The goal of this study is to establish nanoallergens as a versatile platform to study the immunogenicity of potential IgE binding epitopes of major allergen proteins. Therefore, we first developed a synthetic strategy to formulate nanoallergens that can effectively display potential IgE binding epitopes on their surfaces in a multivalent fashion to trigger mast cell degranulation. For this purpose, we selected Ara h2, the major peanut allergy protein, to study because it is known to have immunoreactivity with over 90% of patients who have life-threatening peanut allergies^21^. A thorough evaluation of current literature on Ara h2 IgE binding epitopes revealed eight ubiquitously cited epitopes (Table 1)^21–26^.

**Table 1:** Ara h 2 Epitopes.

#	Sequence	Resides	Notes	References
1	NLRPCEQHLMQKIQRD	38–53	alpha helix 2	Mueller *et al*.^21^. Maleki *et al*.^22^.
2*	ERDPYSP^OH^SQDPYSP^OH^S	79–91		Mueller *et al*.^21^. Stanley, JS^23^. McDermott *et al*.,
3	SDRLQGRQQ	114–123		Mueller *et al*.^21^, Bernard *et al*.
4	RRCQSQLER	28–35	alpha helix 1	Mueller *et al*.^21^, Stanley, JS^23^. Albrecht, M, 2009.
5	HASARQQWEL	15–24		Albrecht, M, 2009.
6	RQQEQQFKRELRNLPQQ	120–136	alpha helix 5	Mueller *et al*.^21^,
7	PQRCDLE	142–148		Mueller *et al*.^21^,
8	CDLEVESGGRDRY	145–157	C terminus of protein	McDermott *et al*.^24^.

*Note: Epitope 2 has two hydroxyproline post-translational modifications that were incorporated into the epitope-lipid conjugate.

**Figure 1:**
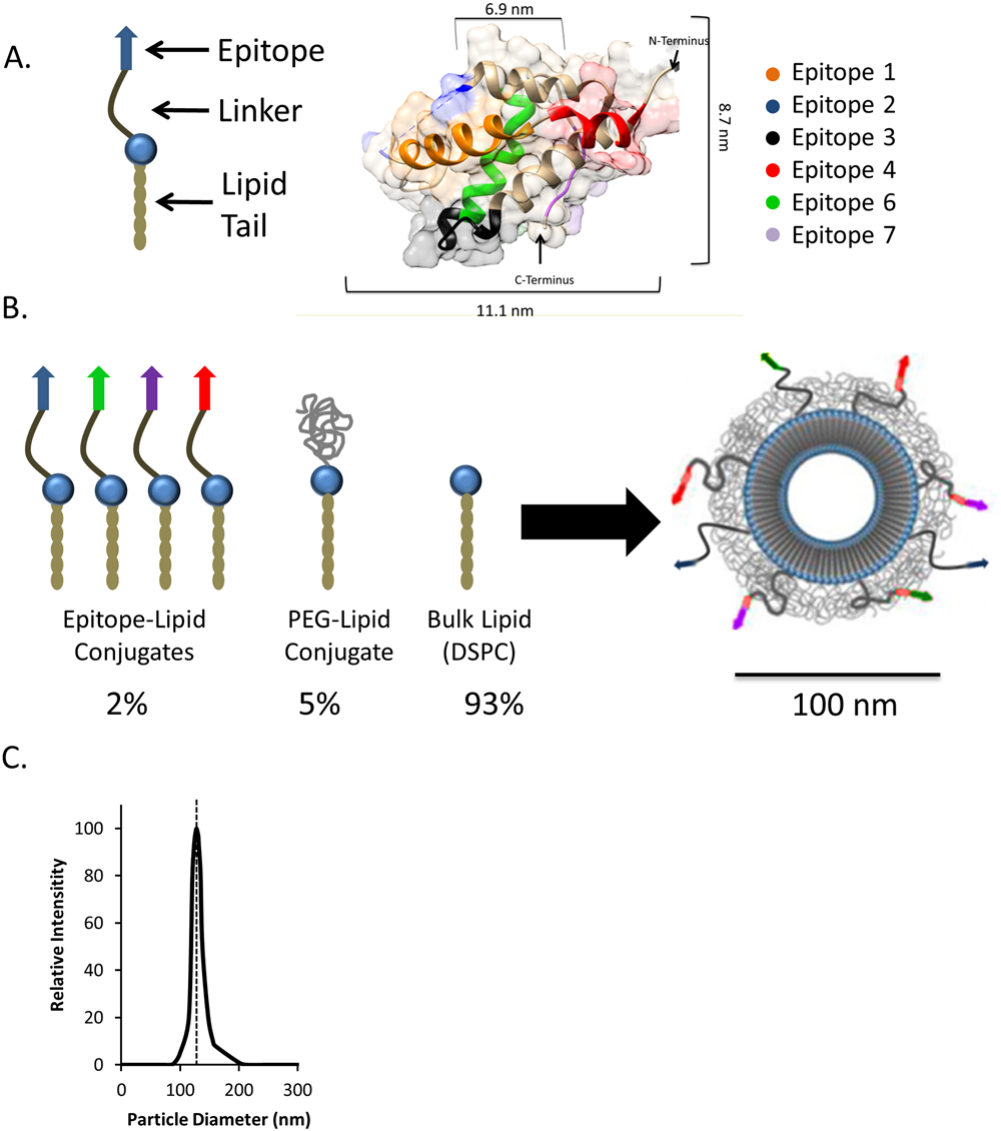
Nanoallergen design, synthesis and characterization. (**A**) Epitope-lipid conjugate design for presenting Ara h 2 epitopes on nanoallergens is shown where epitope is peptide mimetic of IgE binding epitope of Ara h 2. The crystal structure of Ara h 2 with major IgE binding epitopes are color coded. Note that epitope 5 and 8 are not shown due to their presence on the N and C terminus of the protein respectively. (**B**) Schematic of nanoallergen synthesis. Epitope-lipids were synthesized and purified as stated above and then mixed with a PEG-lipid conjugate and DSPC at a ratio of 2:5:93 and extruded to form 100 nm particles. (**C**) Particle sizing of the nanoallergens was determined by DLS. Nanoallergen effective diameter was consistently 128 nm for the nanoallergens. A representative data is shown.

**Figure 2:**
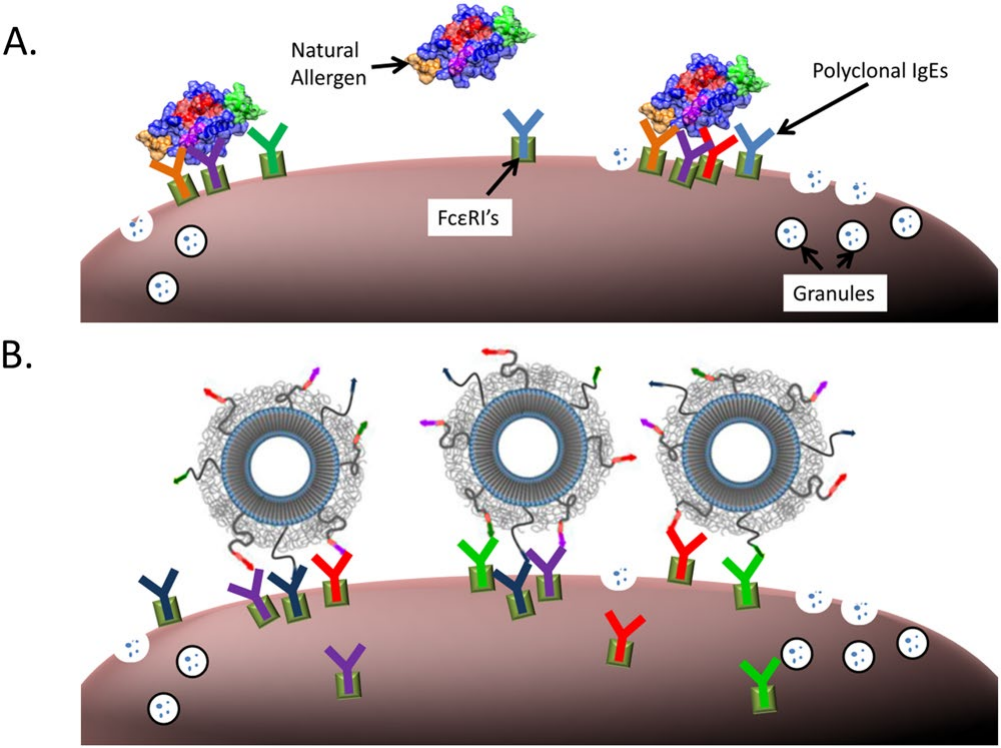
Cartoon representation of nanoallergen triggering degranulation compared to natural allergens. (**A**) Cartoon representation of the crosslinking events induced by the natural peanut allergen protein, Ara h 2 (with different colors labeling potential epitopes on the protein and their corresponding IgEs), leading to mast cell degranulation. (**B**) Nanoallergens are designed to present immunogenic epitopes to trigger crosslinking and degranulation in a similar fashion as natural allergens. This cartoon was adapted from Deak *et al*.^13^.

The potential IgE binding epitopes of Ara h2 from Table 1 were individually presented on the surfaces of nanoallergens using our method of surface modified liposome formation^16–18^. In this approach, epitopes, taken as linear peptide sequences from Table 1, were conjugated to lipid tails to create epitope-lipids, which allows epitope exposure on liposome surfaces with precise control over epitope loading (Fig. 1A)^16, 17^. These epitope-lipids consist of a linear peptide sequence of an IgE binding epitope, a linker region to ensure efficient epitope display on particle surfaces, and a lipid tail to facilitate insertion into liposomal membranes (Figs 1A, S1). Epitope-lipids were individually synthesized using solid phase peptide synthesis (SPPS) (Table S1). Epitope-lipids were then mixed at a 93:5:2 molar ratio of a bulk lipid (DSPC), a PEGylated-lipid conjugate (DSPE-PEG2000) and epitope-lipid, and extruded through membranes to ensure consistent particle size and loading (Fig. 1B). All nanoallergens were loaded with 2% epitope-lipid (by molar ratios for all percentages) unless otherwise noted and extruded through 100 nm pore membranes. The effective diameter of 2% epitope loaded nanoallergens was determined to be 128 nm as was monitored with DLS (Fig. 1C). This increase in size was due to the presence of the PEG coating, as blank liposomes also demonstrated a similar increase in effective diameter (data not shown). Using this design, we predict that epitopes can crosslink epitope specific IgEs bound to FcεRI in a similar fashion as natural allergen proteins allowing us to directly observe a degranulation response to individual epitopes (Fig. 2).

### Validation of the Nanoallergen Platform as a Clinically Relevant *in Vitro* Assay System to Study Degranulation

**Figure 3:**
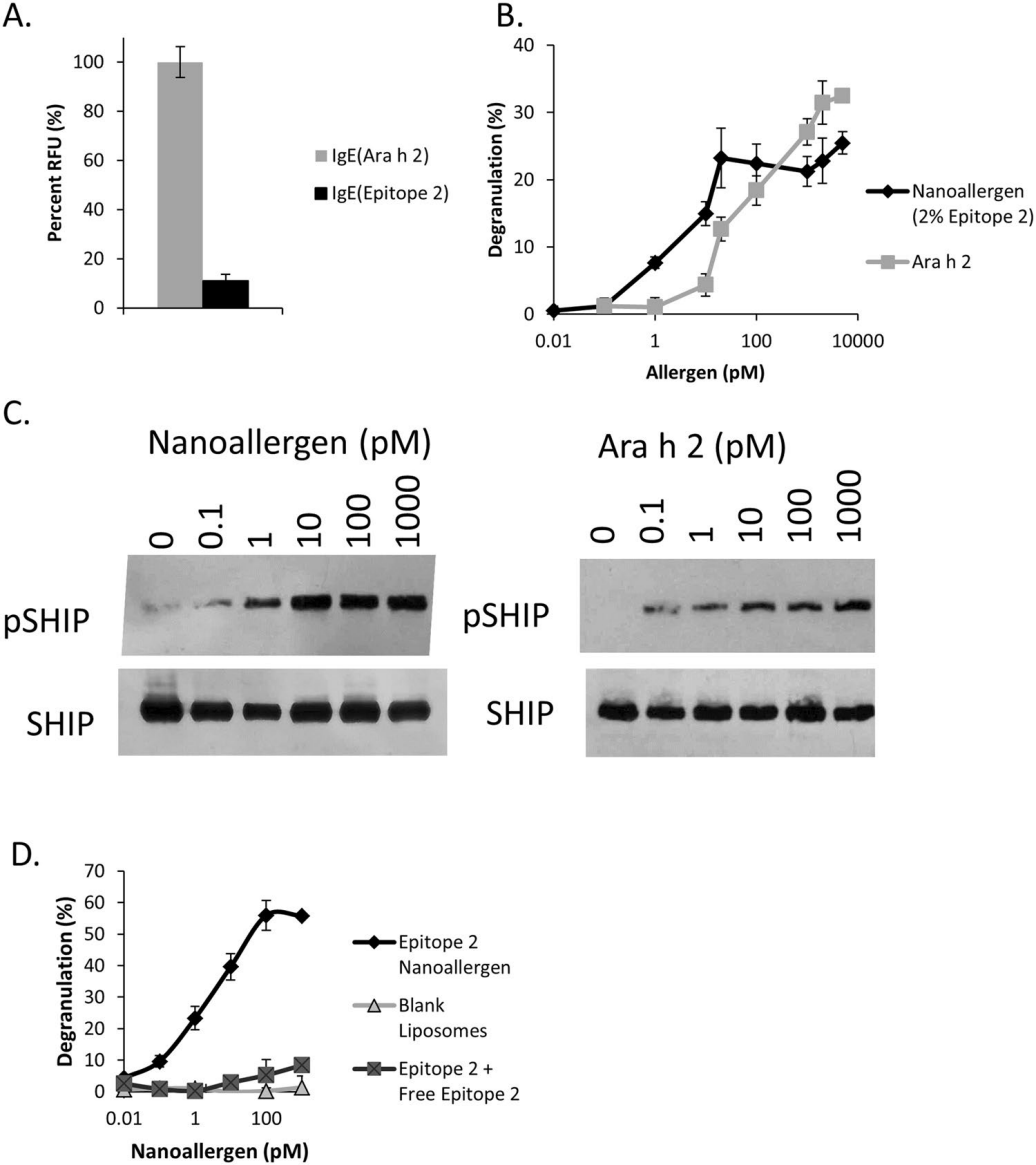
Nanoallergens are designed to present Ara h 2 epitopes and trigger degranulation. (**A**) IgEs specific to Ara h 2 protein and Ara h 2 epitope 2 were identified in sera taken from a peanut sensitive patient using an ELISA assay. Binding to their respective antigens was determined by comparing maximum fluorescence values, which demonstrated that approximately 10% of Ara h 2 IgEs were specific for epitope 2. (**B**) Epitope 2 was loaded into nanoallergens at a 2% concentration and used to stimulate degranulation in RBL-SX38 cells that were primed with 10% serum from a peanut allergy patient. (**C**) Epitope 2 loaded nanoallergens were incubated with RBL-SX38 cells and a western blot analysis was performed on cell lysates to examine SHIP phosphorylation. (**D**) Control experiments performed in the presence of 50 μM free epitope peptide 2 or when blank liposomes were used demonstrated almost complete inhibition of degranulation.

To validate that the nanoallergen platform can be used as an effective assay system to study mast cell degranulation, we engineered nanoallergens presenting the Ara h2 epitope 2 (Table 1). We performed *in vitro* degranulation assays using nanoallergens on a single patient serum to evaluate and optimize the nanoallergen platform in general. To our knowledge, there are no assays currently available that enable the study of how a single epitope induces degranulation using patient sera as the sensitization agent. This is likely due to low sIgEs concentrations and the low affinity of linear epitope mimetics^27^. The nanoallergen platform, however, enables presentation of specific epitopes at high valency on surface of liposomes in order to achieve the proper avidity to overcome these issues and trigger degranulation responses. For our validation study, first we chose epitope 2 of Ara h2 because it demonstrated detectable binding to patient sera IgEs in the ELISA experiments we performed, likely indicating a greater affinity to sIgEs than the other epitopes (Fig. 3A, Fig. S2).

In order to evaluate degranulation using human patient sera, we modified the well-established rat basophilic leukemia (RBL) degranulation assay by using an engineered version of the RBL cell line, RBL-SX38, which was transfected to express the human FcεRI on their surface. We primed these RBL-SX38 cells using sera from a patient with peanut allergies (serum 1) and induced crosslinking using the synthesized nanoallergens. This serum was purchased commercially (Plasma Labs International) and is taken from a patient with a history of peanut allergy reactions and with a high peanut specific IgE level (Table S2). In our assays, we quantified immunogenicity by measuring the maximum degranulation (D_max_) response observed by the nanoallergens and the concentration of nanoallergens that induced half maximal of the degranulation response (EC_50_) (see methods for calculations).

Our experiments performed with nanoallergens loaded with 2% (by molar ratio) of epitope 2 demonstrated significant degranulation response in the RBL-SX38 cells (Fig. 3B). We observed significant degranulation (p < 0.05 when compared to blank liposome control) at low picomolar to nanomolar ranges, which are typical concentrations for very immunogenic allergens (Fig. 3B)^25^. We also observed that nanoallergens activated two intracellular pathways of RBL-SX38 cells in a similar manner as the natural Ara h2 allergen protein using western blot. Both the nanoallergen and Ara h2 protein induced phosphorylation of phosphatidylinositol-3, 4, 5-trisphosphate 5-phosphatase 1 (SHIP), an intracellular hydrolase commonly activated during antigen stimulation in degranulation responses, indicating that both by the nanoallergen and Ara h2 activate IgE mediated signaling pathways (Fig. 3C, see Fig. S3A,B for full length gels)^28, 29^. Additionally, control experiments performed in the presence of excess free epitope 2 peptide demonstrated a near complete inhibition of degranulation induced by the nanoallergens demonstrating that the degranulation was occurring specifically as a result of epitope 2-sIgE crosslinking interactions (Fig. 3D). Furthermore, no degranulation was observed when RBL-SX38 cells not containing human FcεRI (RBL-2H3 cells) were sensitized with serum and challenged or when nanoallergens presenting a scrambled sequence of epitope 2 were used to challenge the cells, further confirming a specific IgE degranulation pathway (Fig. S4). This is consistent with the literature that RBL cells that possess IgG receptors on their surface that share homology with human IgG receptors, have been shown not to trigger degranulation^30^. Finally, to demonstrate the effects of epitope valency on immunogenicity, we performed degranulation assays with nanoallergens synthesized with varying epitope loading (between 0.01–10%, Fig. S5). Taken together, this data confirms that nanoallergens can trigger IgE dependent degranulation responses to IgE binding epitopes using clinical samples.

### Determination of the Immunogenicity of Ara h2 Epitopes in a Clinical Sample using the Nanoallergen Platform

**Figure 4:**
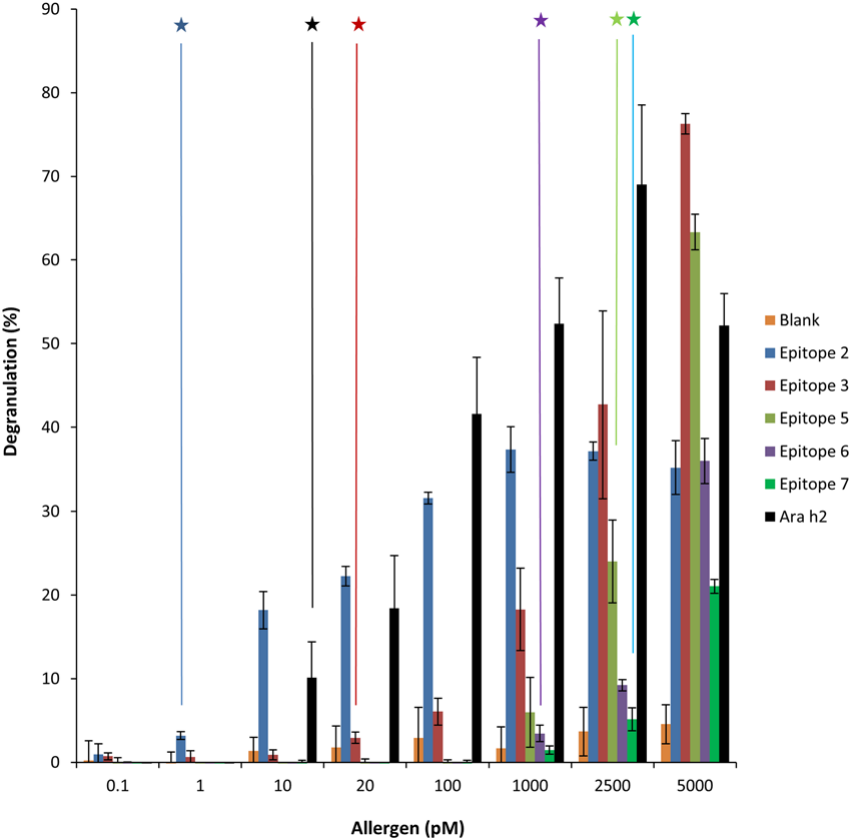
Nanoallergens demonstrate immunogenicity of Ara h2 IgE binding epitopes 1–8 with patient sera. Nanoallergens were loaded with 2% of each Ara h 2 IgE binding epitope and used to trigger degranulation with RBL-SX38 cells primed with 10% serum 1. Colored stars indicate concentration at which degranulation occurred at a significant level above blank liposome control (p < 0.01).

After we confirmed that nanoallergens can be used as an effective assay platform to determine the immunogenicity of allergen epitopes, we sought to rank the relative immunogenicities of each potential IgE binding epitope of Ara h2, obtaining an “immunogenic epitope profile” for this patient. Given the clonal variability of sIgEs for peanuts in patient populations, we assume each patient will have a unique epitope profile. We synthesized nanoallergens that multivalently displayed one of the eight potential epitopes for Ara h2 from Table 1 and tested them using our modified degranulation assay describe above. Five of the eight epitopes (2, 3, 5, 6 and 7) triggered degranulation when presented on nanoallergens while the remaining three epitopes (1, 4 and 8) had little or no response when compared to blank liposome control (Fig. 4, Table S3, Fig. S6). While nanoallergens presenting epitope 2 were able to trigger degranulation at only 1pM concentration, nanoallergens presenting epitopes 3, 5, 6,7 triggered significant degranulation responses in the high picomolar concentrations, which is typical for highly potent allergen proteins (Fig. 4)^31^. Importantly, the nanoallergens presenting epitope 2 were able to trigger significant degranulation at only 1 pM concentration, indicating it is more immunogenic than other epitopes (Table 2, Fig. 4). Additionally, we estimate that approximately 10% of all IgEs directed against Ara h2 in serum 1 might be specific for epitope 2. While IgEs specific for other epitopes were not detectable with ELISA assay, 10% of the ELISA signal for Ara h2 was observed using a labeled version of epitope 2, although an exact IgE ratio would require further evaluation (Fig. 3A). Combined, these results demonstrated the importance of epitope 2 for triggering the allergic response in this particular patient. We hypothesized that the enhanced immunogenicity of epitope 2 was due to the presence of proline hydroxylations at positions 85 and 92 on the primary sequence of Ara h2^32^. To test this hypothesis, we synthesized nanoallergens presenting epitope 2 with and without proline hydroxylations and performed degranulation assays. Our results demonstrated a significant increase in immunogenicity for epitope 2 containing proline hydroxylations, confirming reports in literature (Fig. S7)^33^. Taken together, our study showed that the nanoallergen platform enabled a quantitative measure of immunogenicity for each IgE binding epitope of Ara h2 for this patient.

**Table 2:** Immunoreactivity of Ara h 2 epitopes with serum 1.

Epitope	Immuno-reactivity (Serum 1)
1	−
2	++
3	+
4	−
5	+
6	+
7	+
8	−

### Engineering Nanoallergens to Present Epitope Combinations for More Accurate Allergen Modeling and Determination of Most Critical Epitopes of Ara h2

**Figure 5:**
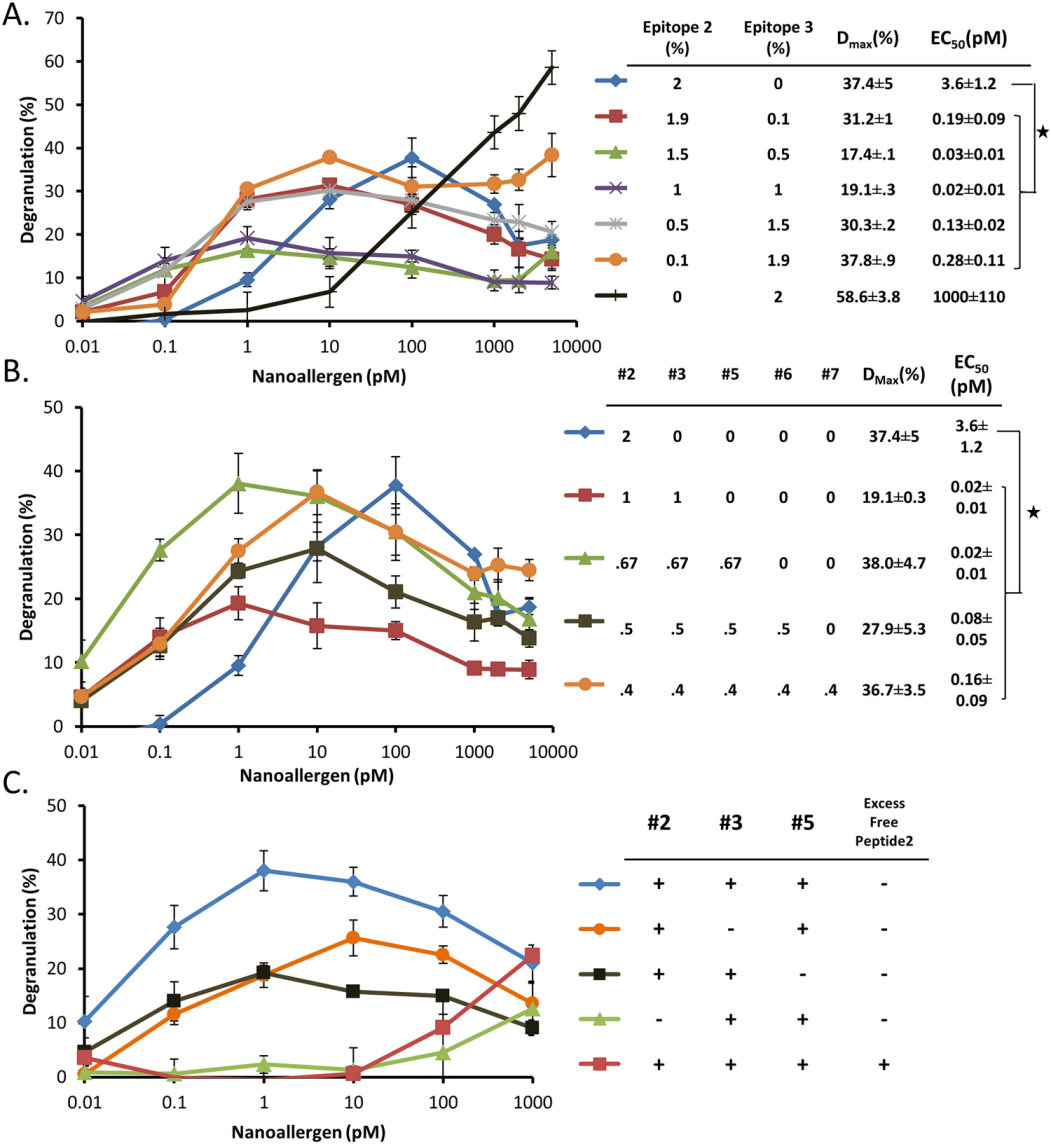
Heterogeneous nanoallergens presenting a combination of Ara h 2 epitopes reveal which epitopes are the most crucial for degranulation. (**A**) Epitope 2 and 3 were mixed at varying ratios in nanoallergen formulations to examine degranulation responses. (**B**) Nanoallergens presenting epitope 2 was combined with other immunoreactive epitopes (3,5,6,7) while maintaining a constant total epitope loading of 2% and used to trigger degranulation. (**C**) A nanoallergen with a mixture of epitope 2,3 and 5 was used to trigger degranulation with epitope 2 or 3 or 5 omitted from the formulation or in the presence of free epitope 2 peptide (50 μM). Stars indicate p < 0.05.

Our nanoallergen platform also allows expression of several epitopes in combination on a single particle, providing a more accurate model system to study immunogenicity. Allergen proteins typically have between 3–8 immunoreactive epitopes which function in combination to provide overall antigen immunogenicity^34^. In order to more accurately simulate these interactions and evaluate the effect of Ara h2 epitopes on overall antigen immunogenicity, we synthesized nanoallergens to present multiple epitopes from Table 1 on the same particle. Importantly, we demonstrated that high and low immunogenic epitopes can act synergistically to increase overall allergen immunogenicity^35^. In light of this, we chose a combination of two different epitopes, a highly immunogenic one and a low immunogenic one based on our results shown in Fig. 4, to observe the impact of low immunogenic epitopes when presented in combination with highly immunogenic epitopes. Specifically, we chose epitope 3 as the representative low immunogenic epitope and epitope 2 as the highly immunogenic epitope due to their significant difference (>200-fold) in EC_50_ (Fig. 4, EC_50_, epitope 2 = 4 pM; EC_50_ epitope 3 = 1000 pM). We synthesized nanoallergens that presented epitope 2 and 3 at various ratios (2/0, 1.9/0.1, 1.5/0.5, 1/1, 0.5/1.5, 0.1/1.9, 0/2%) on the surface of the particle while keeping the total percentage of epitopes at 2%. When the lower immunogenic epitope 3 was introduced at a 0.1% ratio and epitope 2 decreased to 1.9% from 2%, we observed a significant decrease in the degranulation EC_50_ value, contrary to expectations given that there were fewer high immunogenic epitopes (Fig. 5A). More interestingly, all nanoallergen epitope combinations containing epitope 3 (0.1–1.9%) had a decrease in EC_50_ values over nanoparticles presenting only epitope 2 (Fig. 5A). This result suggests that high and low immunogenic epitopes can combine synergistically to trigger more robust degranulation than high immunogenic epitopes alone. This is significant because it demonstrates that often overlooked lower affinity epitopes can significantly alter overall immunogenicity.

To further emulate Ara h2, we synthesized nanoallergens with up to all five of the epitopes that demonstrated any degranulation based on our previous experiment performed with homogenous nanoparticles (epitope 2, 3, 5, 6, and 7, Fig. 4). Given that Ara h2 has a one to one ratio for each epitope, we synthesized the nanoallergen to present an equal ratio of the epitopes on its surface to mimic this. Specifically, we synthesized nanoallergens with epitope 2 (the most immunogenic) and systematically added other epitopes to make five nanoallergen formulations (2; 2, 3; 2, 3, 5; 2, 3, 5, 6; 2, 3, 5, 6, 7) while maintaining the total epitope loading constant at 2%. Our results demonstrated that all nanoallergens that contain one or more epitopes in addition to epitope 2 had decreased EC_50_ values when compared to nanoallergen displaying epitope 2 only (Fig. 5B). The data indicated that nanoallergen presenting a 1:1:1 ratio (0.67% of each) of combination of epitopes 2, 3, 5 was the most immunogenic combination given it had the lowest EC_50_ value and highest D_max_ (Fig. 5B). Given the marked increase in immunogenicity of the nanoallergen presenting epitope 2, 3 and 5 when compared to other combinations, we can conclude that certain combinations with high and low immunogenic epitopes appear to enhance immunogenicity synergistically while others are antagonistic.

To further understand what makes the nanoallergen presenting epitope 2, 3 and 5 so highly immunogenic, we evaluated the contribution of each of these epitopes to the overall immunogenic response. For this, we synthesized nanoallergens by omitting each epitope one at a time and studied degranulation responses. When epitope 2 was omitted from the nanoallergen, the degranulation response dropped to near baseline levels (Fig. 5C). Omitting either epitope 3 or 5, on the other hand, had a minimal effect on EC_50_ value albeit a drop in D_max_ (Fig. 5C) presumably due to intracellular regulatory responses (see below). We also added an excess of epitope 2 as a free peptide in combination with nanoallergens presenting epitope 2, 3, and 5 to selectively inhibit epitope 2-sIgEs interactions. Our results showed again an almost complete drop in degranulation responses (Fig. 5C). These experiments combined illustrate that certain low immunogenic epitopes synergistically enhance the overall immunogenicity when presented in combination with epitope 2.

### Nanoallergens Reveal the Impact of SHIP Regulatory Responses to Specific Epitope Combinations

**Figure 6:**
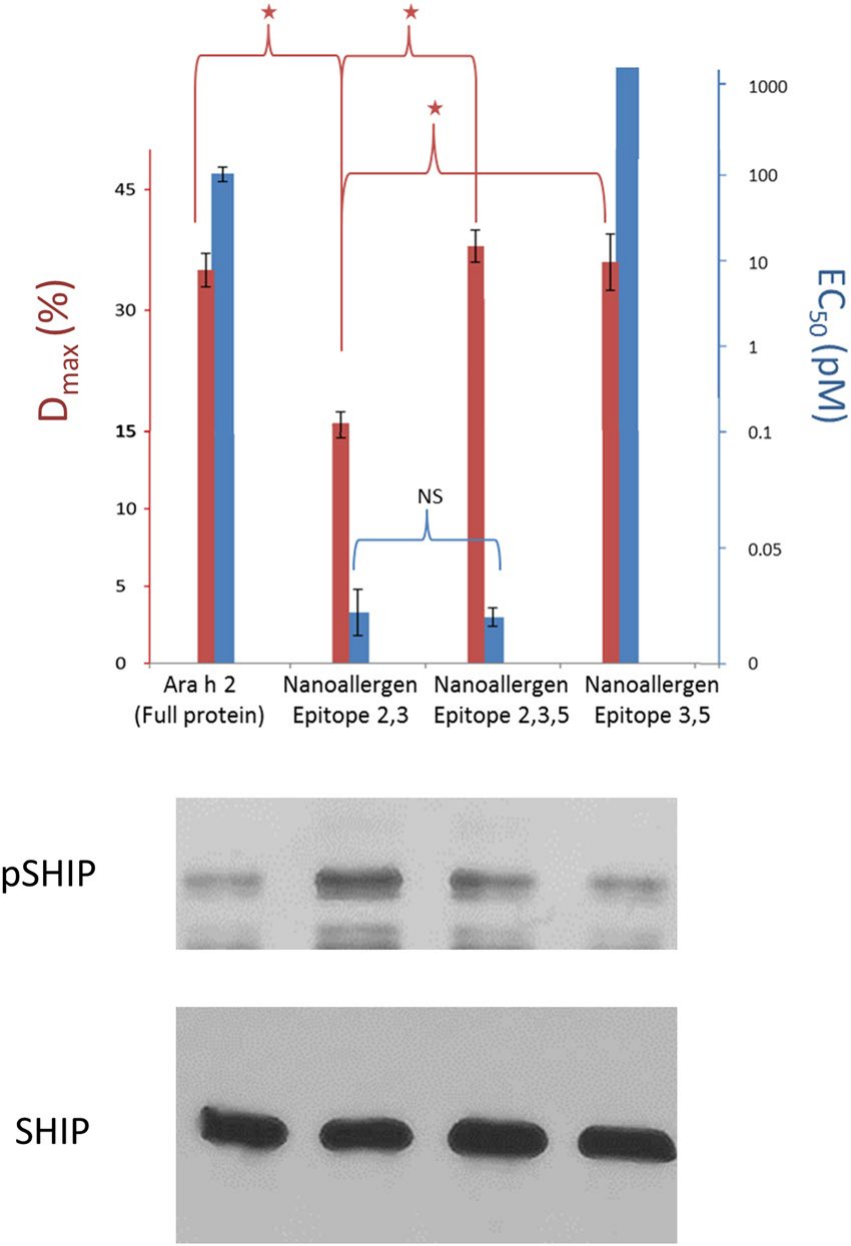
SHIP phosphorylation varies for different nanoallergen formulations. Western blots were performed using RBL-SX38 cells sensitized with patient sera 1. Ara h 2 or nanoallergen formulations as indicated (loaded with 2% total epitope-lipid at 1:1 ratios or 1:1:1 ratios) were added at 100 pM. Graph indicates both EC_50_ value (on right axis, colored blue) and maximum degranulation observed with nanoallergen formulation (on left axis, colored red). Gels were cropped for clarity, see Fig. S3 for full length blots. Stars indicate p < 0.05, note that for epitope 3,5 nanoallergen EC_50_ > 1000 pM.

One of the most interesting results that emerged from the studies performed with combination nanoparticles was a significant drop in maximum degranulation response despite lower EC_50_ values for several nanoallergen combinations, for example nanoallergens presenting epitope 2 and 3 (Fig. 5A,B). We hypothesized that the drop in maximum degranulation is due to formation of long lasting crosslinking events that then cause overstimulation and an excessive activation of the SHIP pathway, which typically has an inhibitory effect on degranulation^35^. To evaluate this, we analyzed SHIP phosphorylation (pSHIP) in RBL-SX38 cells stimulated with nanoallergens and compared this data to EC_50_ values for degranulation responses. As demonstrated in Fig. 6, SHIP phosphorylation is increased for nanoallergens that have a low D_max_ value, such as nanoallergens presenting epitope 2 and 3. This suggests an increase in pSHIP for certain epitope combinations, tempering the D_max_ value, but not the EC_50_ value of the degranulation response. While further evaluation would be necessary to confirm this, this result suggests that an ideal composition of both high and low epitopes on antigens are necessary for triggering high degranulation responses, which avoids relegation by intracellular regulatory cascades. More importantly, this experiment demonstrates the proof of principle that nanoallergens can be used to elucidate the complex mechanistic aspects of degranulation.

### Analysis of Immunogenicity Trends in a Clinical Population by using the Nanoallergen Platform

**Figure 7:**
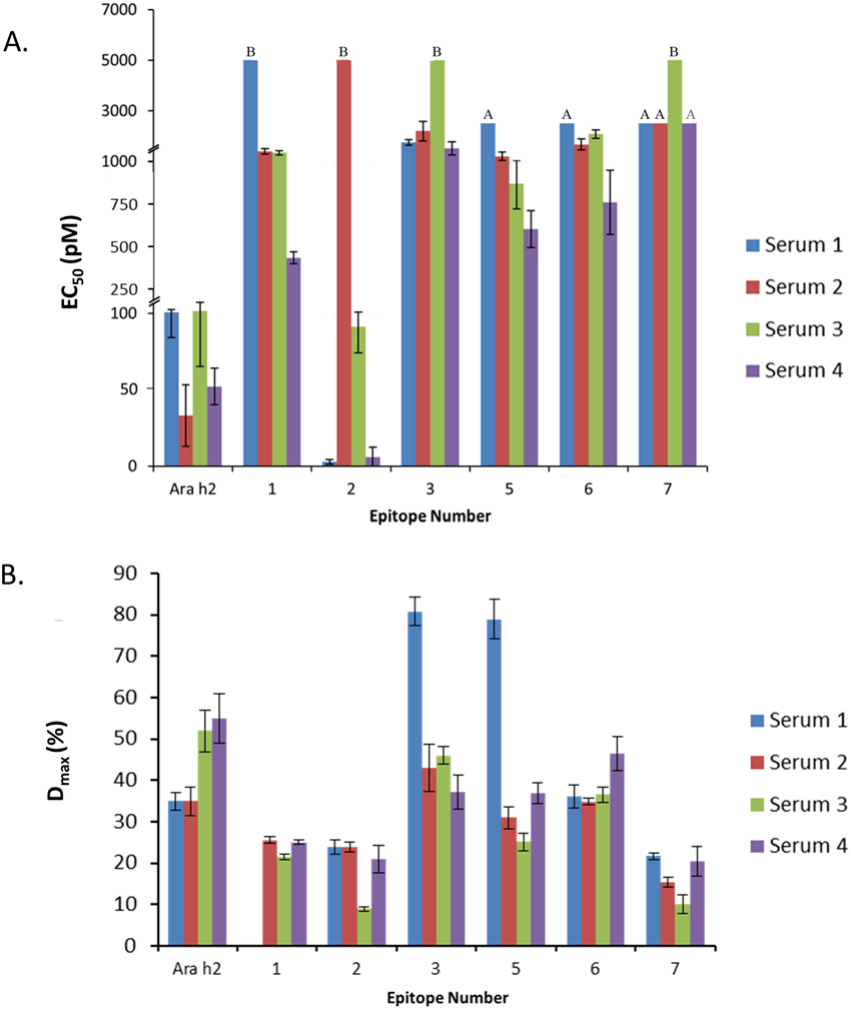
Nanoallergens reveal crucial epitopes in a clinical population. Four patient sera were tested for degranulation response with nanoallergens presenting Ara h2 epitopes. (**A**) EC_50_ values of the degranulation assays are shown. Note that epitope 4 and 8 did not demonstrate any degranulation in any of the patient sera. A’s indicate a EC_50_ values between 5000–2500 pM, B’s indicate a EC_50_ values > 5000pM. (**B**) D_max_ values for degranulation assays.

Thus far these studies demonstrated the utility of the nanoallergen platform to dissect the immunogenicity of epitopes alone and in combination and develop an immunogenic epitope profile for an individual patient. Next, we evaluated the broader use of the nanoallergen platform in a clinical setting by assessing a larger population of allergy patients. While four patients is not sufficient to make definitive claims about trends in patient populations, it does demonstrate how nanoallergens can be used to identify the immunogenic epitope profile for each patient. We acquired sera from four peanut allergy patients with histories of severe clinical reactions. We confirmed these sera to have high sIgE concentrations for peanut and Ara h2, and that they stimulated degranulation in response to Ara h2 in RBL-SX38 cell assays and binding to Ara h2 and epitope 2 with ELISA (Tables S2, S3, Fig. S8). Then, using the nanoallergen platform, we evaluated these patient sera for their immunoreactivity to all eight Ara h2 IgE binding epitopes (epitopes 1–8, Table 1). All four samples similarly demonstrated moderate responses to epitope 5 and 6 (500 < EC_50_ < 2000 pM) and low responses to epitope 7, and no responses to epitope 8 (EC_50_ > 2000 pM, Fig. 6, Fig. S6). The clinical population had variability as demonstrated by responses to nanoallergen presenting epitope 1 and 3 but the variability is most clearly seen in epitope 2 (ranging from no response to very high responses, Fig. 7). These results demonstrate how nanoallergens could be used with larger clinical populations to establish trends in critical immunogenic epitopes and potentially provide personalized assessment of these variable allergen epitopes, leading to more effective patient diagnosis and allowing the development of personalized therapies to target immunogenic epitopes.

To further examine the utility of nanoallergens to assess any allergen protein epitopes, these experiments were also repeated with epitopes taken from Ara h 6, which is another critical peanut allergen protein^36^. We assessed seven potential IgE binding epitopes of Ara h 6 using the nanoallergen platform in a similar fashion, presenting each epitope individually on the nanoallergen surface and triggering degranulation using patient sera (Tables S4, S5)^37^. Two out of the four sera (1,and 4) demonstrated highly immunogenic responses to Ara h 6 epitope 3, similar to the EC_50_ values seen from Ara h2 epitope 2 with these patients (Table S6, Fig. S9). Given that there is a 60% sequence homology between Ara h2 and Ara h 6 and an established homology between these two epitopes, this result suggests that the same sIgEs are potentially cross-reactive to several peanut proteins, a result which has also been demonstrated in literature^38^. These results further validate nanoallergens as an effective platform for developing immunogenic epitope profiles in a clinical setting for a wide variety of allergy proteins, and further experimentation with a larger number of clinical samples would more broadly strengthen our conclusions.

## Discussion

Here we described the development and assessment of nanoallergens, a nanoparticle based platform that can multivalently display single or multiple epitopes with precise control over stoichiometric ratios for patient specific study of allergen epitope immunogenicity. Our studies establish nanoallergens as a reliable platform that enables assessment of each individual allergen epitope’s immunogenicity for a single patient by triggering degranulation with a single epitope at a time. Importantly, nanoallergens are a significant improvement over current epitope analysis methods, such as ELISA microarrays, because they reveal the importance of epitopes with weaker immunogenicity and are able to correlate a linear epitope directly to a cellular response.

Nanoallergens can also be used to study mechanistic aspects of degranulation. One of the key findings of this study is that nanoallergens that displayed a combination of high and low immunogenic epitopes can trigger more degranulation at a hundred-fold lower concentration than the nanoallergen displaying high immunogenic epitope alone for one of the patient samples, despite the reduction in the density of the high immunogenic epitope on particle surface. This finding is very significant as it demonstrates that the often-overlooked lower affinity epitopes can drastically alter overall immunogenicity and potential clinical responses of allergy proteins. We speculate this increase in immunogenicity as owing to the increased avidity of the nanoallergen presenting high and low immunogenic epitopes. Avidity of a multivalent entity depends on both the monovalent affinities of the moieties as well as the total number of binding interactions, hence the valency of the epitopes on nanoallergens as well as of the IgEs on cell surface carry significance. Since, immunogenicity depends upon both EC_50_ and D_max_, the inclusion of low affinity epitopes can improve the overall avidity of the nanoallergen-IgE/FcεRI interaction by providing additional anchorage points for binding, thereby decreasing the EC_50_. Likewise, additional epitope-IgE/FcεRI interactions can also increase the size of the FcεRI cluster, thereby increasing D_max_, as is seen by certain heterogeneous nanoallergens (Fig. 5A)^39^. We conclude that heterogeneous nanoallergens with the proper ratio of high and low immunogenic epitopes, while able to retain long particle residence times on the surface of the cell due to the presence of high immunogenic epitopes, are able to transiently form large FcεRI receptor clusters and do not initiate inhibitory SHIP activation; and therefore possess the highest immunogenicity. While it is not possible to draw conclusions concerning the specific high and low affinity epitopes for all patients from this one sample owing to the clonal variance in IgEs for patients, we do believe that the interaction between any high and low affinity epitopes will be relatable between patients. Furthermore, this technique does not take into account conformational epitopes on allergen proteins which are important for overall allergen immunogenicity. However, other groups have recently identified linear peptide mimetics of IgE binding conformational epitopes, incorporation of which would be straightforward into nanoallergens^40^. Additionally, nanoallergens are larger than most allergen proteins (≈10 nm versus 100 nm diameter), which would likely increase the size of potential FcεRI clusters, making nanoallergens intrinsically more immunogenic than natural allergens, therefore making direct comparisons difficult.

Despite these limitations, this platform provides quantitative measures to evaluate the immunogenicity of any individual epitope for a specific patient by isolating discrete epitopes and directly correlating them to a biological response. Our nanoallergen platform enabled the establishment of patient immunogenic profiles. This type of analysis was not possible with the currently existing methods and provides an invaluable tool that might aid diagnostics or future therapy options. As the study demonstrates, nanoallergens are also very adaptable and can be applied to any known allergen protein and could potentially be used to identify and evaluate new IgE binding epitopes. Hence, this study presents nanoallergens and established the proof of principle that this platform can elucidate factors determining allergen immunogenicity for individual epitopes.

Overall, the results of the present study emphasize the significance of nanoallergen platform as an advanced and physiologically relevant method for detailed characterization of allergen epitopes and degranulation responses in allergy research. Furthermore, we demonstrate how nanoallergens can be used to sketch out the immunogenic profile for a patient by presenting allergen epitopes separately as well as in combinations to elucidate their immunogenicities. The direct evaluation of epitope immunogenicity through tracking degranulation responses gives the nanoallergen platform a marked advantage over multi-epitope ELISA microarrays currently used to evaluate IgE binding epitopes. Nanoallergens can also take advantage of this microarray format, where nanoallergens could be used to display a library of potential epitopes, and degranulation assays or other immunological assays could provide the readout. Furthermore, it has been demonstrated in literature that immunoreactivity to certain epitopes is correlated with severe physiological reactions in a patient^41^. Therefore, a nanoallergen defined immunogenic profile could potentially be used to more accurately predict the severity of an individual’s prospective reaction to an allergen and provide an advantage over current diagnostic allergy tests. While any diagnostic value for nanoallergens will require further clinical evaluation with large patient populations, this initial study is important for characterizing the potential scientific and diagnostic features nanoallergens offer. Taken together, this study introduces nanoallergens as a versatile platform for studying allergic reactions and for providing clinically useful patient specific information regarding allergen epitopes that could improve diagnosis, enable targeted designs for future therapeutics and allow for personalized treatment options for patients.

## Materials and Methods

### Materials

We purchased NovaPEG Rink Amide resin, HBTU [2-(1*H*-benzotriazol-1-yl)-1,1,3,3 tetramethyluroniumhexafluorophosphate], all Fmoc conjugated amino acids and BSA (Bovine Serum Albumin) from EMD Biosciences. DIEA (*N*,*N*-diisopropylethylamine), TFA (trifluoroacetic acid), Triisopropylsilane (TIS), hydrazine, Cholesterol, Dichloromethane, 2-proponol, ACN(acetonitrile), ethanol, all Kaiser test reagents, G418 salt and Bovine serum albumin (BSA, tween 20 and piperidine were purchased from Sigma. We obtained DMF (dimethylformamide) (>99.8%), chloroform, penicillin, L-glutamine and Eagle’s Minimum Essential Media from Thermo Fisher. DSPC (1,2-distearoyl-*sn*-glycero-3-phosphocholine), DSPE-mPEG2000 (1,2-distearoyl-*sn*-glycero-3-phosphoethanolamine-N-[methoxy(polyethylene glycol)-2000] (ammonium salt)), membranes and all mini extruder components were purchased from Avanti Polar Lipids (Alabaster, Al, USA). Fmoc-EG_6_-OH was purchased from Quanta Biodesign. DiD fluorescent dye (3H-Indolium, 2-(5-(1,3-dihydro-3,3-dimethyl-1-octadecyl-2H-indol-2-ylidene)-1,3-pentadienyl)-3,3-dimethyl-1-octadecyl-, perchlorate) and fluorescein 5(6) isothiocyanate was purchased from Invitrogen. Western Blot reagents, gels and equipment were obtained from Bio-Rad. Goat anti-rabbit IgG (ab6721), and anti-human IgE (ab99834) were purchased from Abcam. RIPA buffer and phosphatase inhibitor-I was purchased from Boston Bioproducts (Boston). Anti-rabbit-HRP IgG (cat# 111-035-003) was purchased from Jackson ImmunoResearch. All human serum samples were purchased from PlasmaLab International (Everett, WA). Anti-SHIP conjugated beads (sc-8425 AC), Anti-SHIP antibodies (sc-8425) and anti phospho-tyrosine (p-tyr) antibodies (PY99) were purchased from Santa Cruz Biotechnologies. Tyrodes Buffer was made as previously described^12^.

### Epitope Lipid Synthesis and Purification

The most important component of a nanoallergen particle is the epitope-lipid conjugate. We typically synthesize these molecules using solid phase synthetic methodologies, and using a design approach that was developed in our laboratory^16, 17^. Throughout the article, in order to avoid confusion, all peptide mimotopes and peptide-linker-lipid conjugates originating from corresponding allergen IgE binding epitope sequences are simply referred to as epitopes. The epitope, when as part of the nanoallergen assembly, consists of three moieties: linear peptides of Ara h2 epitope sequences, an ethylene glycol (EG) linker of exact repeating units of EG for a desired length, and two hydrocarbon (C16) tails to facilitate the molecule’s anchoring into lipid bilayer of the liposome (Table 1, Fig. 1A). To maximize the display efficiency of the epitope, the EG linker can be varied in length and oligolysine residues can be incorporated in the design of the peptide-linker-lipid conjugate. The results of several experiments performed to optimize these design parameters (results not shown) helped determine that an ethylene glycol linker of 18 units (three EG_6_ spacers) and three lysines provide the optimal features for epitope presentation (Fig. S1). We synthesized peptide-linker-lipid conjugates of all eight Ara h2 epitopes using standard Fmoc solid phase peptide synthesis (SPPS) chemistry on NovaPEG Rink Amide resin (Table S1). The procedure is described previously^34^. A sample peptide-linker-lipid conjugate is shown in Fig. S1. Lipid hapten molecules were purified using 1200 Agilent RP-HPLC using a semi-preparative Zorbax C3 column. A two phase water and 70/20/10 IPA/ACN/water mix was used for purification with a gradient of 60–100% IPA mix over 10 minutes at a flow rate of 3 mL/min. Peptide-lipid conjugates were purified using a Zorbax C18 column, using a two phase water/ACN system with a gradient of 20–50% ACN in 10 minutes. Absorbance peaks at 220 nm and 280 nm were collected and verified for purity with analytical injections (>95%). The product was confirmed using a Bruker microTOF II mass spectrometer.

### Nanoallergen Preparation

Liposomal nanoallergens were prepared using a procedure as previously described^16, 17^. Nanoallergens were prepared the day of triplicate experiments and used for all three independent experiments. The liposomes were comprised of between 0.1–10% (mole percent) of peptide-linker-lipid conjugate, 5% polyethylene glycol 2000-lipid conjugate to inhibit non-specific interactions, and the remaining mole percent of the molecules in liposomes was a bulk phospholipid (DSPC). Unless otherwise stated, the nanoallergens in this study typically included a 2% peptide-linker-lipid conjugate, and were 100 nm in diameter. Briefly, DSPC, mPEG-2000-DSPC, cholesterol, and peptide-linker-lipid conjugates were dissolved in chloroform, lyophilized for 30 minutes following rotate evaporation, rehydrated in PBS at 60 °C, and then extruded through a 100 nm polycarbonate filter (Avanti).

### Particle Characterization

The size of liposomes were confirmed using DLS analysis via the 90 Plus nanoparticle size analyzer (Brookhaven Instruments Corp., Long Island, NY), using 658 nm light observed at a fixed angle of 90° at 20 °C.

### Cell Culture

RBL-2H3 cells were purchased from ATCC. RBL-SX38 cells were a generous gift from Dr. Jean-Pierre Kinet from Harvard University. RBL-SX38 were cultured in Minimum Essential Media (Gibco) with 10% fetal bovine serum (Gemini BioProducts, Sacramento CA) and 1.2 mg/mL of G418 salt (Sigma) as previously described^42^. RBL-2H3 cells were cultured in same media, however without the addition of G418.

### Degranulation Assays

Degranulation assays were performed as previously described using nanoallergens as the allergen^43^. RBL-SX38 cells were plated into 96 well dishes for 24 hours and then incubated with 10% of human sera in cell culture media for an additional 24 hours prior to the degranulation assay.

### Degranulation Calculations

Percent degranulation was calculated as previously decribed^12^. Degranulation maximum (D_max_) and EC_50_ values were calculated using Origin 7 software using Hill’s curve fit for sigmoidal curves using percent degranulation values taken from experiments.

### ELISA Assay

A high binding 96 well plate was incubated with anti-Human IgE in Carbonate-Bicarbonate buffer (Sigma) at a concentration of 3 nM for 16 hours at 4 °C. Plate was washed with washing buffer (PBS with 0.5% tween 20) and then blocked with blocking buffer (5% BSA in PBS with 0.1% tween 20) for 1 hr at room temperature. Plate was washed with automatic plate washer (AquaMax 2000), then varying concentrations of either Ara h2-Biotin (Indoors Biotechnologies) or FITC-peptide 2 conjugate in blocking buffer for 1 hour. Plate was washed again and then either streptavidin-HRP (1:5 000 dilution) or anti-FITC HRP IgG (1:5000 dilution) was added in blocking buffer. After another washing step, Amplex Red substrate was added (Invitrogen) and plate fluorescence was read at 5 minute intervals according to manufacturer’s instructions.

### Western Blot

Stimulated RBL cells were analyzed for intracellular activity with a western blotting technique previously described for expression of p-SHIP^35^. RBL-SX38 cells were plated at approximately 0.5 × 10^6^ cells per mL into a 3 mL dish for 24 hours followed by incubation in 10% patient serum in cell culture media for another 24 hours. Cells were then washed with Tyrodes buffer and incubated with Tyrodes for 30 minutes at 37 °C. Ara h2 or nanoallergens were then added at varying concentrations and incubated at 37 °C for 3 minutes. Cells were washed with ice cold PBS, then incubated in RIPA with phosphatase inhibitor lysis buffer, scrapped and sonicated for one minute intervals over the course of 30 minutes on ice. Lysates were then spun down at 15,000 RPM for 10 minutes and their protein concentration determined by Bradford Assay. Laemmli buffer and PBS were added to all lysates so that their protein concentration was 0.5 mg/mL or would be 0.5 mg/mL prior to immunoprecipitation. Samples were boiled for 5 minutes; centrifuged and 20 uL of each was added to a 10% SDS-PAGE gel. Samples ran on the gel for 1 hr, were transferred to nitrocellulose paper for 1.5 hrs, and blocked with 5% BSA in tris buffered saline with 0.1% tween 20 (TBS-T)for 1 hr. Primary antibodies were added at the manufacturer’s suggested dilutions in blocking buffer (either 2000X or 500X), washed with TBS-T, then appropriate secondary antibodies with HRP conjugates were added according to manufacturer’s dilutions (typically 1:10,000). The membranes were washed, and incubated in with Clarity™ Western ECL Blotting Substrate (Bio-Rad) for 5 minutes. Bands were exposed onto Kodak Chemiluminescence Film for times ranging from 1–30 minutes.

### Statistics

All error bars represent the standard deviation of three independent replicates of cells. All p values were calculated using a one tailed student’s t test.

### Ethics Approval

All human samples used in this study were purchased from a third party vendor using anonymous donors.

### Availability of Data

All data referenced in this study is included in the manuscript or supplemental file. All supplemental data is available online.

## Electronic supplementary material

Supplementary Information
