# Supplementary material for: Determination of Crucial Immunogenic Epitopes in Major Peanut Allergy Protein, Ara h2, via Novel Nanoallergen Platform
Source: Sci Rep. 2017 Jun 21;7:3981. doi: 10.1038/s41598-017-04268-6 (PMC5479826; doi:10.1038/s41598-017-04268-6)
Supplement: Supplementary file 1 — Supplementary Information [file 41598_2017_4268_MOESM1_ESM.pdf]

Supplementary Information for:

Determination of Crucial Immunogenic Epitopes in Major Peanut Allergy Protein, Ara h2, via Novel Nanoallergen Platform

Peter E. Deak[†], Maura R. Vrabel[†], Tanyel Kiziltepe[†,‡] and Basar Bilgicer[†,‡,§,·]

[†]Department of Chemical and Biomolecular Engineering, University of Notre Dame, Notre Dame IN 46556

[‡]Advanced Diagnostics and Therapeutics, University of Notre Dame, Notre Dame IN 46556

[§]Department of Chemistry and Biochemistry, University of Notre Dame, Notre Dame IN 46556

[·] Correspondence:

171 Fitzpatrick Hall
Notre Dame, IN 46556-5637
Tel: 1 574 631 1429
fax: 1 574 631 8366
e-mail: bbilgicer@nd.edu

A.

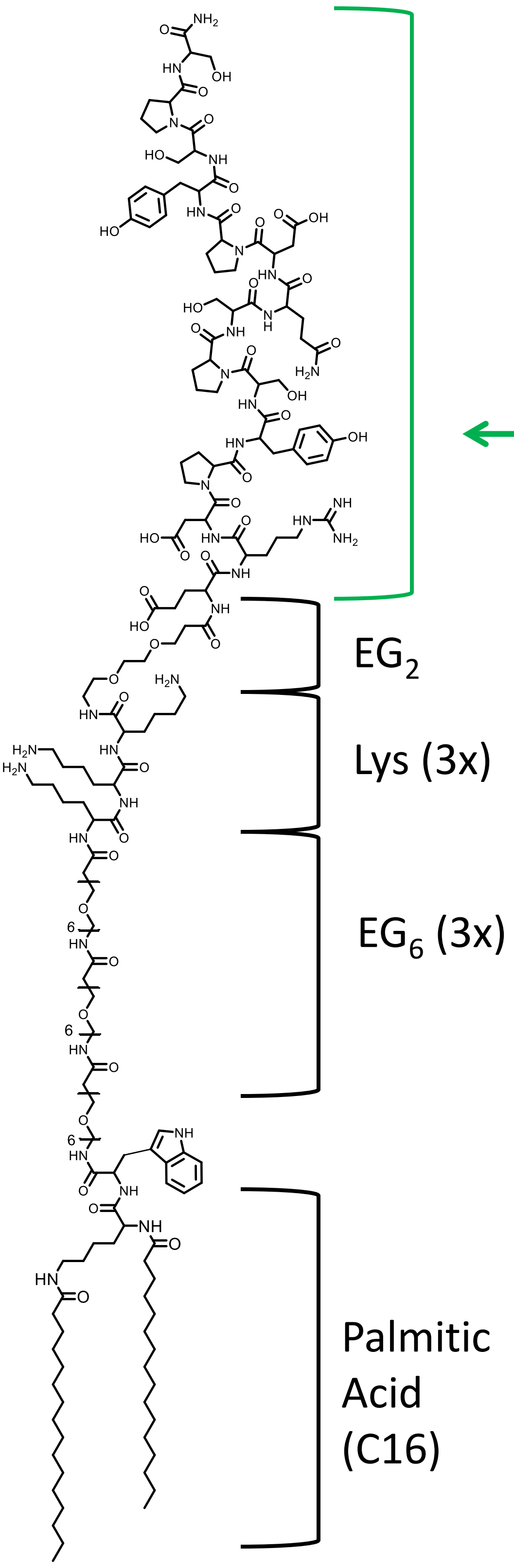

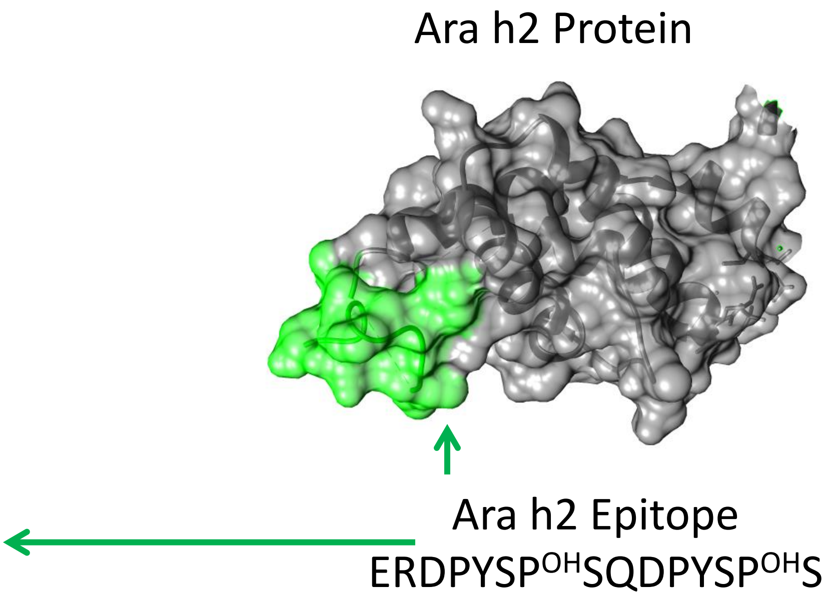

Figure S-1. Schematic of epitope-lipid conjugates . A linear peptide sequence of the peanut protein Ara h2 (residues 79-91) was attached to a lipid using the schematic given. The epitope (shown in green) is shown in context with the native Ara h2 protein (PDB: 3OB4). Note that the schematic was used for all other epitope-lipids.

Table S-1. Listing of Ara h 2 IgE binding epitopes-lipid conjugates.

Epitope-Lipid	Exact Mass (g/mol)	Purity (%)	Immuno-reactivity (Serum 1)
1	4330.64	>95%	None
2	4094.33	>95%	High
3	3425.16	>95%	Low
4	3498.23	>95%	None
5	3563.21	>95%	Low
6	4563.78	>95%	Low
7	3182.02	>95%	Low
8	3820.28	>95%	None

Table S-2. Listing of patient sera and their ImmunoCAP results for peanuts.

Serum Number	Peanut IgE (kU/L)
1	84.4
2	99.3
3	50
4	124.5

Table S-3- EC_{50} values and maximum degranulation values for Ara h 2 and 2 % loaded Ara h 2 epitope nanoallergens

Serum #	1				2				3				4			
Peptide #	EC_{50} (pM)	Error	Max(%)	Error	EC_{50} (pM)		Max(%)	Error	EC_{50} (pM)	Error	Max(%)	Error	EC_{50} (pM)	Error	Max(%)	Error
Ara h2	104	20	35	2.1	33	20	35	3.5	115	50	52	5	52	12	55	6
1	>5000		0		1400	107	25.6	0.9	1340	85	21.5	0.7	430	35	25	0.5
2	3.6	1.2	24	1.7	>5000		24	1.2	91	17	8.9	0.5	5.9	6.6	21	3.3
3	1000	110	80.8	3.4	2200	390	43	5.6	>5000		46	2.1	1512	260	37.2	4.1
5	>2500		79	4.9	1200	170	31	2.6	870	150	25.1	2.1	600	110	37	2.5
6	>2500		36.1	2.7	1670	220	34.9	0.9	2080	170	36.6	1.9	760	190	46.4	4.1
7	>2500		21.7	0.8	>2500		15.3	1.2	>5000		10.1	2.3	>2500		20.4	3.6

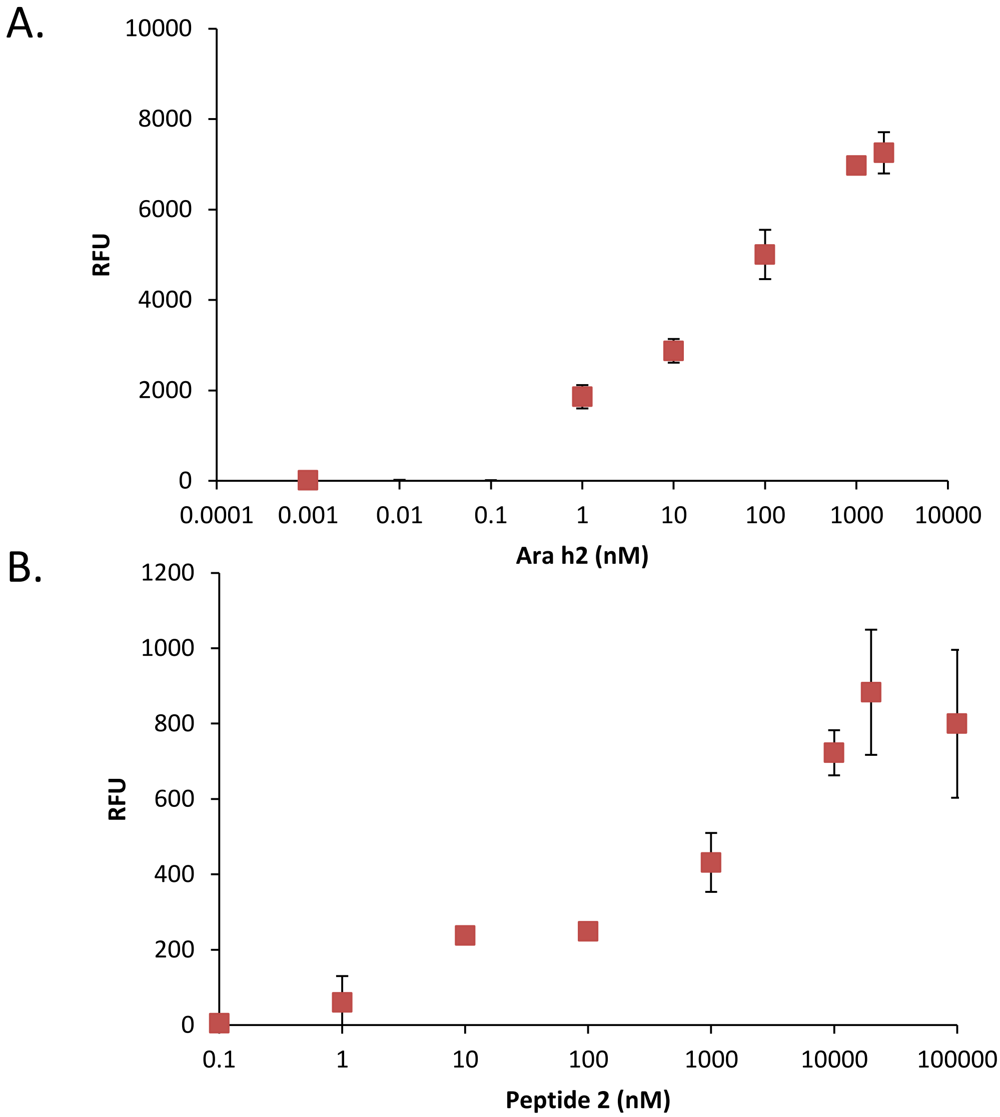

Figure S-2. ELISA binding assay with patient sera. (A) Patient serum 1 was added to a 96 well plate coated with anti-IgE IgG, washed and then either Ara h 2-Biotin or (B) FITC-tagged epitope 2 peptide and the binding was observed using ELISA. Ara h2- EC_{50}= 40 ± 27 nM; Epitope 2- EC_{50}= 465 ± 200 nM

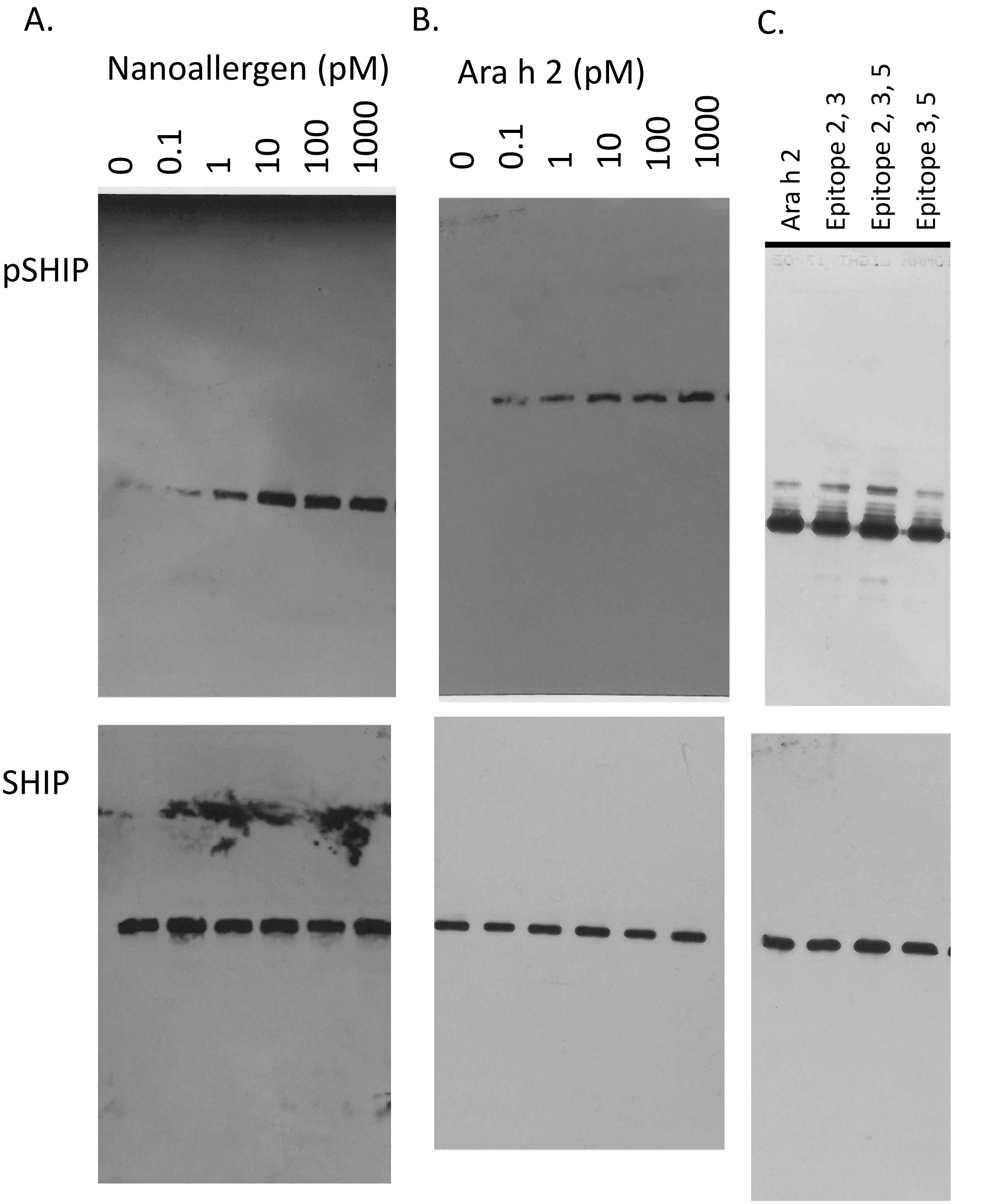

Figure S-3. Full length SHIP blots used in this study. pSHIP shown on top, SHIP at bottom. Note that SHIP-1 protein (MW=133 kDa) appeared between 150 and 100 kDa markers for all gels, with a slight increase in MW for pSHIP.. (A). 2% epitope 2 presenting nanoallergen in Figure 3C, (B) Ara h 2 protein in Figure 3C, (C) multiple nanoallergen formulation and Ara h 2 control in Figure 6. Note bands appearing below pSHIP as possible antibody targeting of SHIP or contamination.

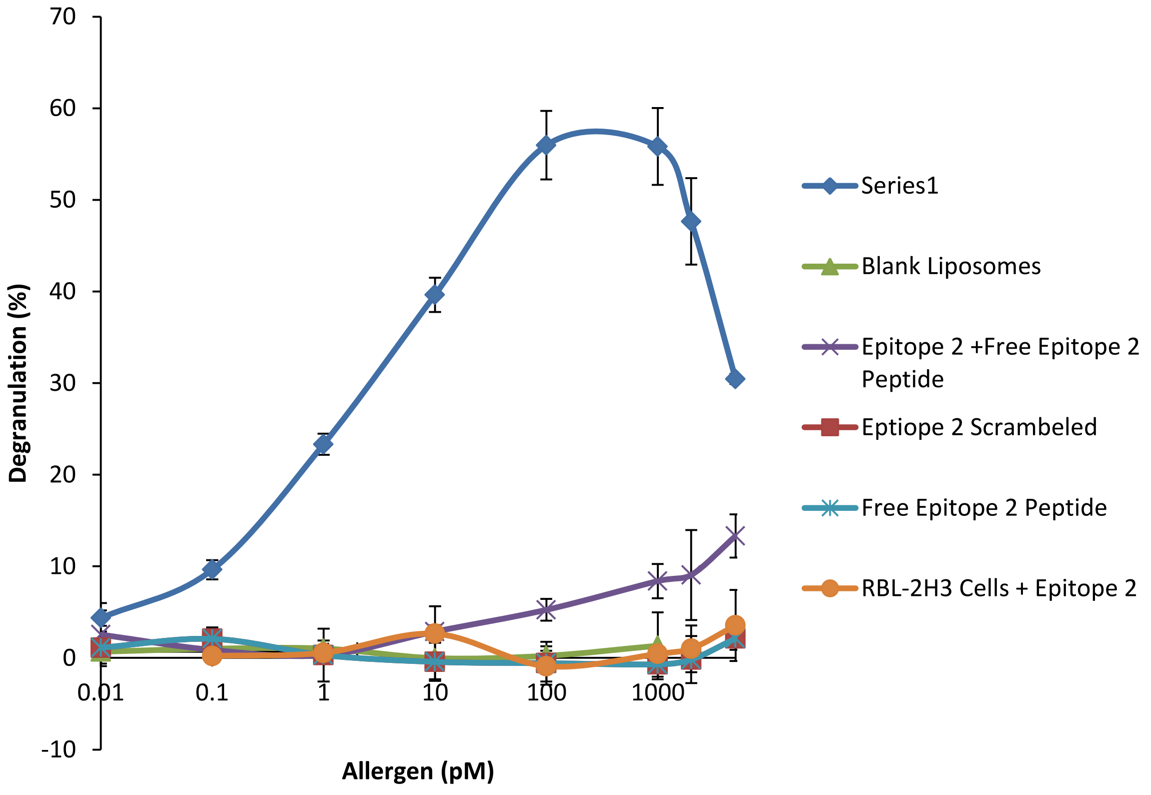

Figure S-4. Nanoallergens demonstrate specificity, as their degranulation response with RBL-SX38 cells can be prevented with addition of 100 μM of free peptide 2 (in purple). Likewise, blank liposomes (in green), liposomes loaded with 2% of epitope 2 sequence that was scrambled (in red) and free epitope 2 alone (in light blue) do not cause a degranulation response. Likewise, cells not expressing the human FcεRI (RBL-2H3 cells) do not demonstrate degranulation when primed with patient serum 1 and incubated with 2% loaded epitope 2 nanoallergens.

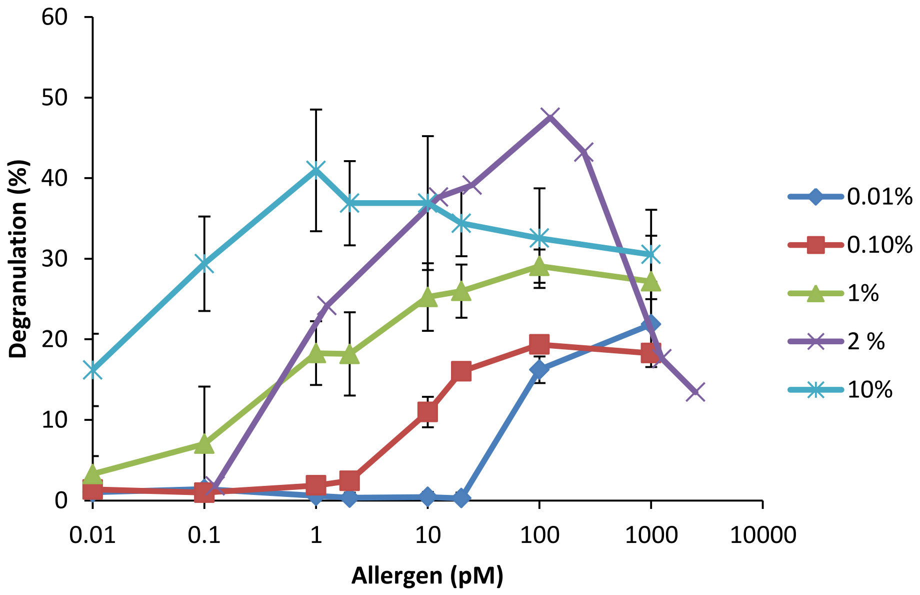

Figure S-5. Nanoallergens can be formed with various epitope loading of epitope 2 and used to trigger degranulation of RBL cells primed with 10% serum 1.

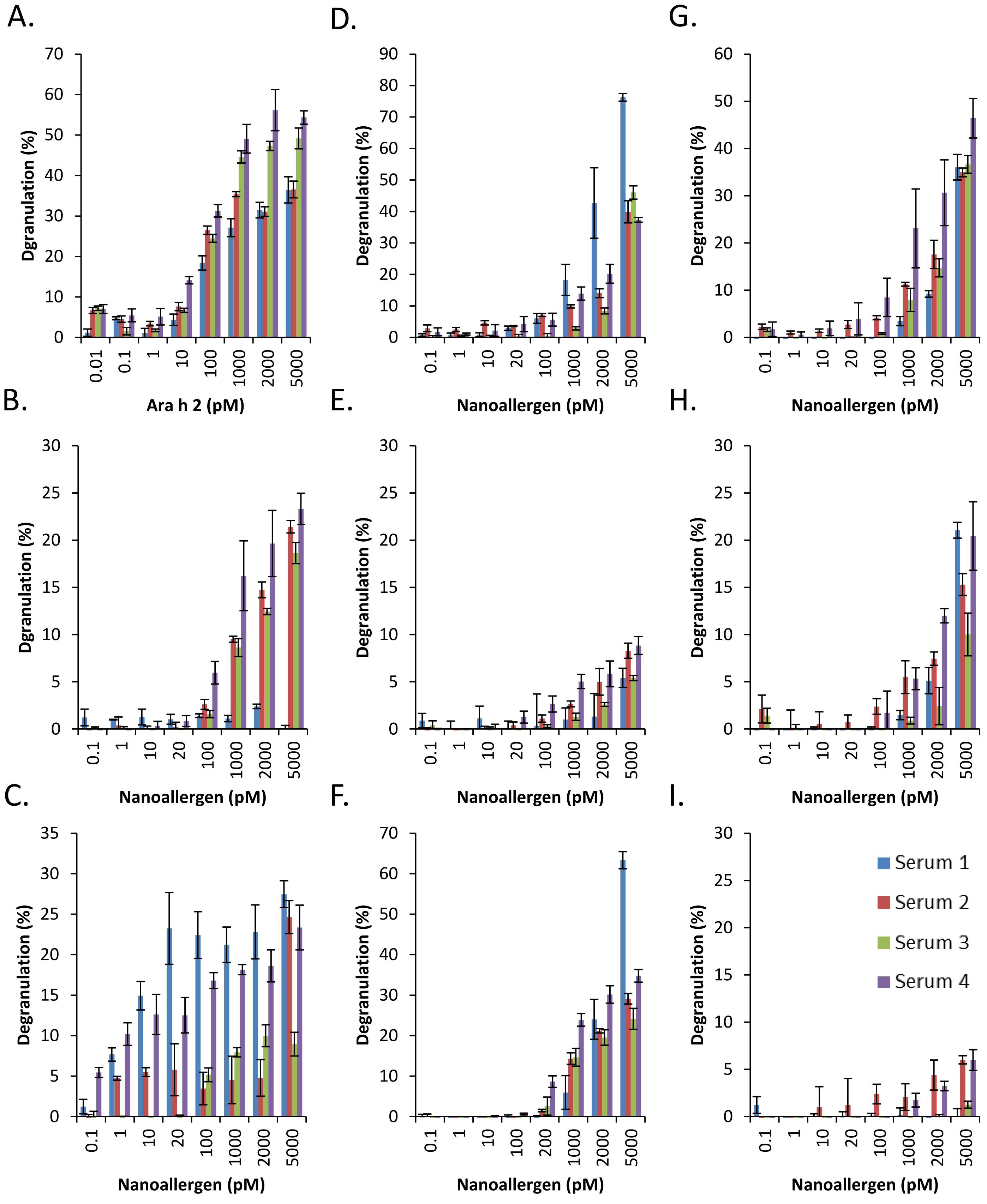

Figure S-6. Raw data for all patient sera/epitope combinations for Ara h 2. Note that 2% epitope loading was used for all assays. (A) is for Ara h 2 protein, while B-I demonstrate epitope 1-8 respectively.

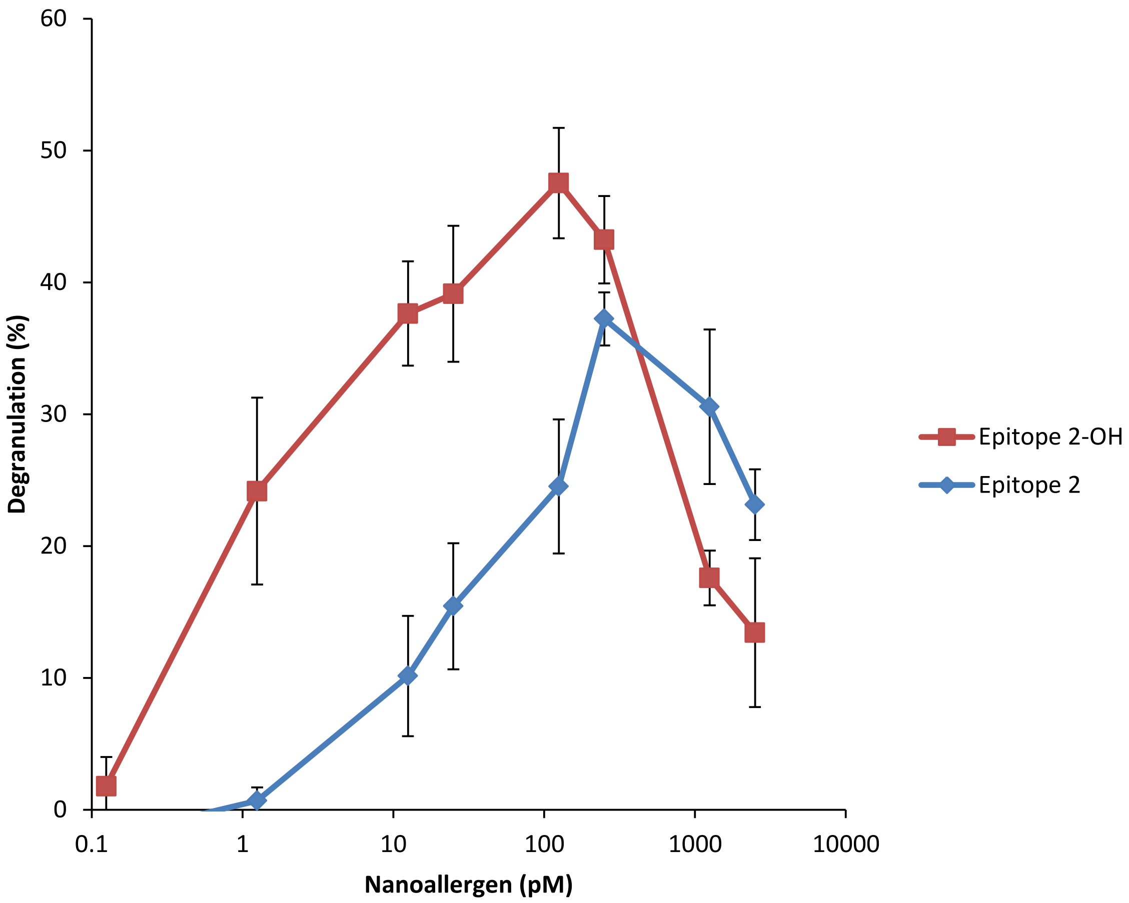

Figure S-7. Epitope peptide 2 demonstrates an increase in response when proline hydroxylations are present. Epitope 2- lipid conjugate was made with and without the addition of proline hydroxylations and loaded at 2% in nanoallergens. Epitope 2, EC_{50} = 21.5 ±8.8 pM; Epitope 2^{OH}, EC_{50}= 0.75±1.5 pM ; p<0.01

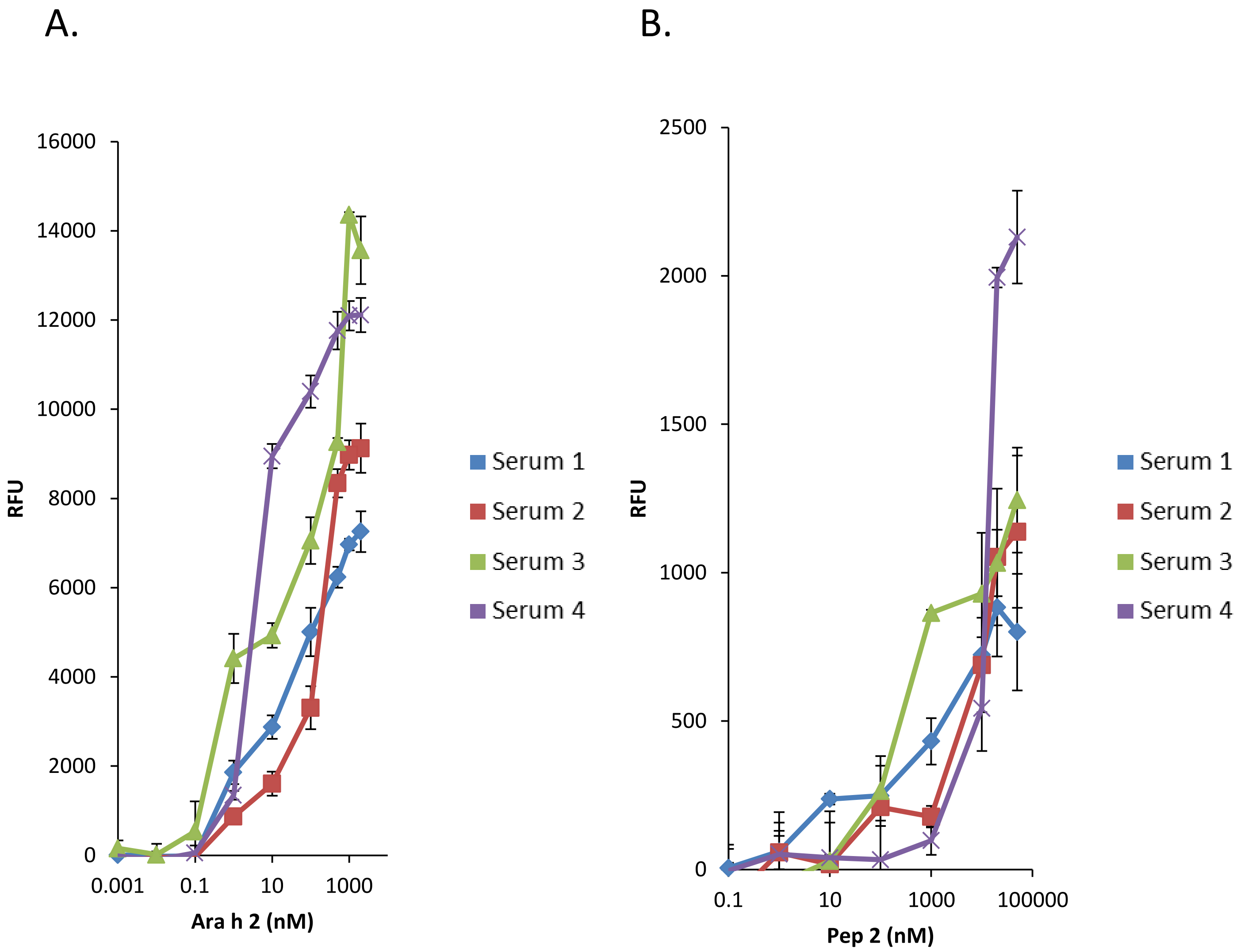

Figure S-8. ELISA binding data for additional sera. (A) Ara h 2 or (B) free epitope peptide 2 was used to bind patient IgE's.

Table S-4: Ara h 6 Epitopes

Epitope#	Sequence	Number	Notes
1	MRRERGRGGDSSSS	24-37	
2	KPCEQHIMQRI	45-55	Homology to ara h2 epitope 11
3	YDSYDIR	35-68	similar to ara h2 epitope 2
4	CDELNEMENTQR	82-93	Homology to ara h2 epitope 10
5	CEALQQIMENQCD	97-109	
6	KRELRMLPQQ	120-129	Homology to ara h2 epitope 7
7	CNFRAPQRCDLDV	130-142	Homology to ara h2 epitope 8

Table S-5. Listing of Ara h 6 IgE binding epitopes-lipid conjugates taken from Ostu *et al*.[18]

Epitope-Lipid	Exact Mass (g/mol)	Purity (%)	Immuno-reactivity (Serum 1)
1	3947.37	>95%	Moderate
2	3733.34	>95%	Mild
3	3110.93	>95%	High
4	3804.23	>95%	Mild
5	3832.45	>95%	Mild
6	3665.35	>95%	Low
7	3728.33	>95%	Mild

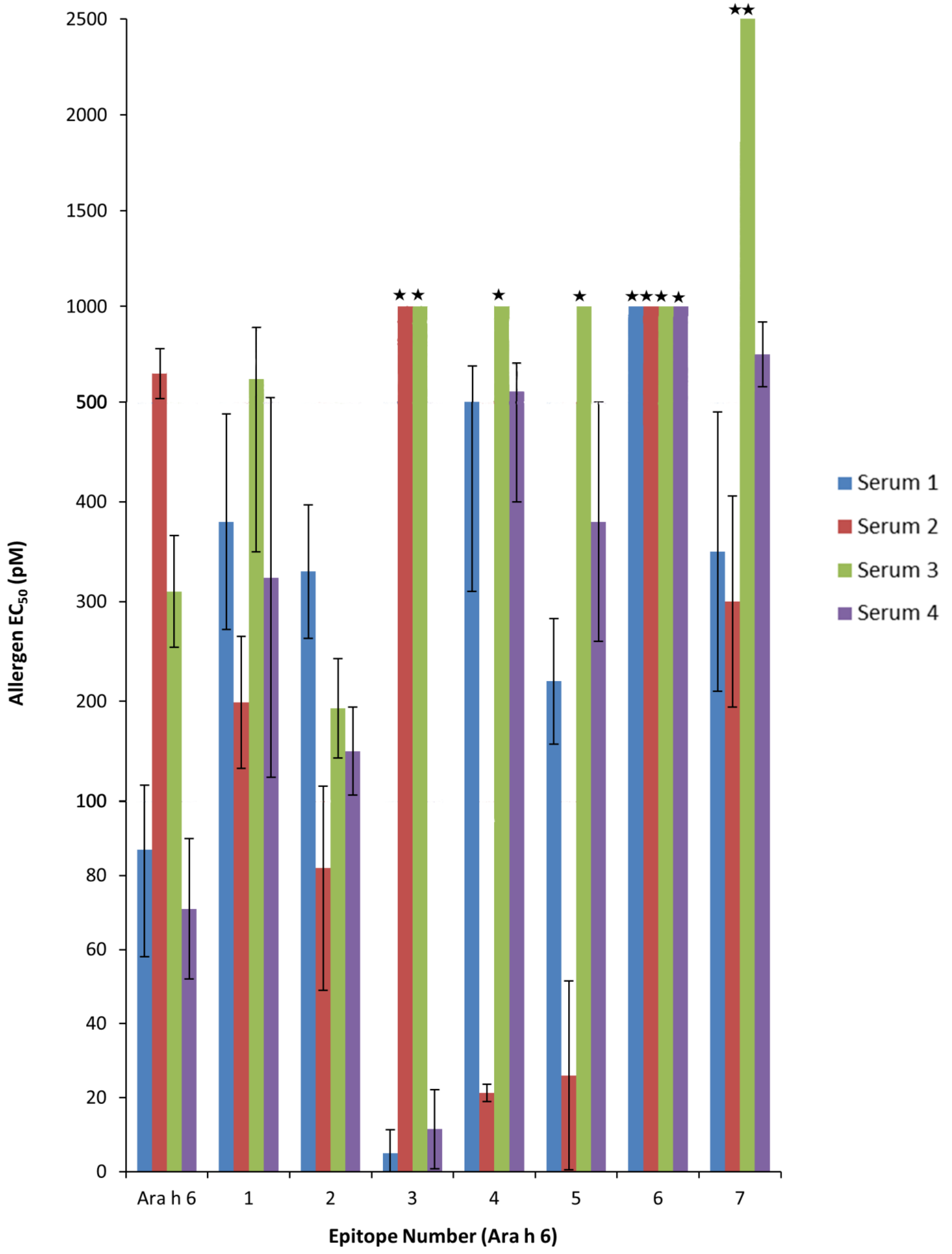

Figure S-9. EC_{50} data for all patient sera/epitope combinations for Ara h 6. Note that 2 % epitope loading was used for all assays.

Table S-6- EC_{50} values and maximum degranulation values for Ara h 6 and 2 % loaded Ara h 6 epitope nanoallergens

Serum #	1				2				3				4			
Peptide #	EC_{50} (pM)	Error	Max(%)	Error	EC_{50} (pM)		Max(%)	Error	EC_{50} (pM)	Error	Max(%)	Error	EC_{50} (pM)	Error	Max(%)	Error
Arah6	87	29	80	5.5	650	130	73	5	310	56	61	3	71	19	91	5
1	380	108	50	4	199	66	60	4.2	620	270	38	4.8	324	200	52	8.8
2	330	67	73	3.1	82	33	78	6.2	193	50	62	3.6	150	44	70	4.2
3	5	6.4	50	7.1	>1000		42.3	4.3	>1000		40.7	2.3	11.5	10.7	47	6.32
4	500	190	25.8	2.1	21.3	2.3	9.6	0.26	>1000		11.3	1.2	550	150	36.2	2.5
5	220	63	18.8	4.43	26	25.5	13	2	>1000		8.9	0.8	380	120	31.7	2.6
6	>1000		30.8	3.8	>1000		33.5	4.7	>1000		17.1	6.5	>1000		18.8	2.5
